# A Literature Review on Optimizing Study Strategies in Medical Education: Insights From Exam Scores and Study Resources

**DOI:** 10.7759/cureus.74034

**Published:** 2024-11-19

**Authors:** Gabriella Morey, Valeria C Morey, Taylor Gruman, Thura Al-Khayat

**Affiliations:** 1 Department of Medical Education, Nova Southeastern University Dr. Kiran C. Patel College of Allopathic Medicine, Davie, USA; 2 Department of Biological Sciences, Florida International University, Miami, USA; 3 School of Medicine, Nova Southeastern University Dr. Kiran C. Patel College of Allopathic Medicine, Fort Lauderdale, USA; 4 Department of Medical Education, Nova Southeastern University Dr. Kiran C. Patel College of Allopathic Medicine, Fort Lauderdale, USA

**Keywords:** academic performance, academic success, medical board study resources, medical education, nbme exams, study methods, study strategies, usmle exams

## Abstract

Medical school exams, like those by the National Board of Medical Examiners (NBME) and the United States Medical Licensing Examination (USMLE), assess essential knowledge and skills for safe patient care, essential for student advancement and securing competitive residencies. Understanding the correlation between exam scores and medical school performance, as well as identifying trends among high scorers, provides valuable insights for both medical students and educators. This review examines the link between study resources and NBME exam scores, as well as psychological factors influencing these outcomes. The focus is on identifying key factors, particularly study resources, that can improve medical student test scores. This literature review synthesizes research from the past 15 years on teaching methods, study resources, and academic success in medical education. While foundational knowledge and study techniques are widely agreed upon, there are gaps in evaluating specific approaches, alternative materials, and the integration of new educational technologies. Key findings from our review include the effectiveness of practice questions and spaced repetition for long-term retention, as well as the impact of prior academic performance and study strategies (e.g., question bank use) on Step 2 Clinical Knowledge (CK) scores. Factors like time management, self-testing skills, and structured preparation programs are crucial to success. Commercial resources like First Aid, UWorld, Boards and Beyond, and Pathoma are noted for their comprehensive support in exam preparation. Future research may offer insights to further optimize study strategies and improve performance.

## Introduction and background

Graduate medical school exams, particularly those written by the National Board of Medical Examiners (NBME) and the United States Medical Licensing Examination (USMLE), are designed to assess the knowledge and skills required for safe and effective patient care [1,2]. Scoring well on these exams is vital, not only for progressing through medical school but also for securing competitive residency placements [1]. Previous research suggests a direct relationship between academic performance in medical school and Step scores; thus, understanding trends among high scorers can help medical students score higher [1].

In modern times, commercially available board resources have become increasingly popular tools for students preparing for clinical-rotation-specific NBME subject exams as well as USMLE Step 1 and Step 2 Clinical Knowledge (Step 2 CK). Some of the resources we came across when searching the literature and speaking with medical students at Nova Southeastern University Dr. Kiran C. Patel College of Allopathic Medicine (NSU MD) are included in Table 1.

**Table 1:** Comprehensive Review Resources for USMLE Preparation. USMLE: United States Medical Licensing Examination; CK: Clinical Knowledge; PDF: portable document format; NBME: National Board of Medical Examiners

Resource	Definition
First Aid for the USMLE [2]	A comprehensive review of high-yield topics and concepts, mnemonics, and essential facts. Included in this resource is a host of learning tools, from key facts and mnemonics to full-color illustrations and proven test-taking strategies. Individual books exist for Step 1, Step 2 CK, and Step 3 [2]
UWorld	An online question bank mimicking the style and difficulty of standardized medical exams, followed by detailed explanations for each answer. The content included in this resource serves as a study aid built by experienced, practicing physicians [3]
Boards and Beyond (B&B), Physeo, Lecturio	These resources are video series discussing various topics essential for the foundation of medical knowledge. B&B [4] includes more than 440 videos and more than 2,300 USMLE-style questions, as well as progress tracking, custom quizzes and playlists, and slide PDFs. Physeo [5] is a comprehensive study containing lectures with integrated flow charts, tables, mnemonics, images, and questions. Lecturio [6] is an online learning platform for medical students with a vast library of video lectures and interactive study materials, covering a wide range of medical topics, including anatomy, physiology, biochemistry, and pharmacology
Pathoma and SketchyMedical	These resources are known for their focus on pathology and microbiology, respectively, using visual aids and concise explanations as reinforcement. Pathoma [7] is a study resource primarily for USMLE Step 1 and preparing for third-year clerkships continuing lectures that cover all of the high-yield pathology points, from a basic mechanistic approach to physiologic integration. SketchyMedical [8] is an interconnected video platform of visual aid stories and recurring symbols to help students learn and reinforce tough concepts of microbiology, pharmacology, and other topics
NBME practice exams	Divided into several forms, the NBME [9] provides official practice exams as a method of gauging student readiness. They are designed by NBME for medical schools to assess students' understanding of basic and clinical sciences in specific content areas

Not only is there a diversity in the types of resources available, but different study strategies may also be highly influential in the success of medical students. Table 2 lists some strategies.

**Table 2:** Key Strategies and Methods for Effective Medical Study. USMLE: United States Medical Licensing Examination

Study strategy	Definition
Active learning [10]	Methods such as flashcards (e.g., Anki) and teaching others can enhance retention and understanding. This method of learning aims at an approach to instruction in which students engage the material they study through reading, writing, talking, listening, and reflecting
Practice questions [10]	Practice questions are a useful resource to identify knowledge gaps by reviewing explanations. Question banks not only identify knowledge gaps but also show medical students if the material from lectures was properly understood [10]. Practice questions can sometimes become an active learning tool by allowing students to actively recall and integrate the concepts they have learned to problem-solve and answer questions
Integrative learning [11]	Integrative learning is a form of medical education that combines different resources and learning modalities, such as textbooks, videos, and practice questions. This process of making connections among concepts and experiences with the aim of applying information and skills to novel and complex issues or challenges develops through high-impact practices like intensive research activities, international experiences, service, and community-based learning [12]
Self-assessment and feedback [13]	Practice exams as well as feedback from mentors or peers help students track their progress and adjust study strategies. Effective and regular feedback reinforces good practice, promotes self-reflection, and motivates the learner to work toward their desired outcome [13]. Feedback can be implemented in a medical student’s training at every level, whether it is a self-assessment reflecting gaps in knowledge or a mentor evaluating the student’s clinical skills
Study schedules [14]	Study schedules may be more of a strategy, but they can be a resource that can provide students with consistent progress and adequate coverage of topics. A solid medical school study schedule helps students master effective study techniques through the organization and application of these study methods in a timely manner. Schedules work by enhancing academic performance and ensuring readiness for critical exams such as USMLE Step 1 and USMLE Step 2 [14]
Balanced lifestyle [15]	A balance between studying and personal well-being is crucial; sleep, exercise, and breaks can improve cognitive function and prevent burnout. A healthy lifestyle, a way of living that reduces the likelihood of severe illness or early death, includes regular physical activity, better sleep patterns, improved dietary habits, and decreased feelings of anxiety. A recent study found that unhealthy lifestyle factors, such as lack of physical activity, inadequate sleep, poor dietary choices, smoking, and mental health issues such as anxiety, have a negative impact on academic performance [15]

**Figure 1:**
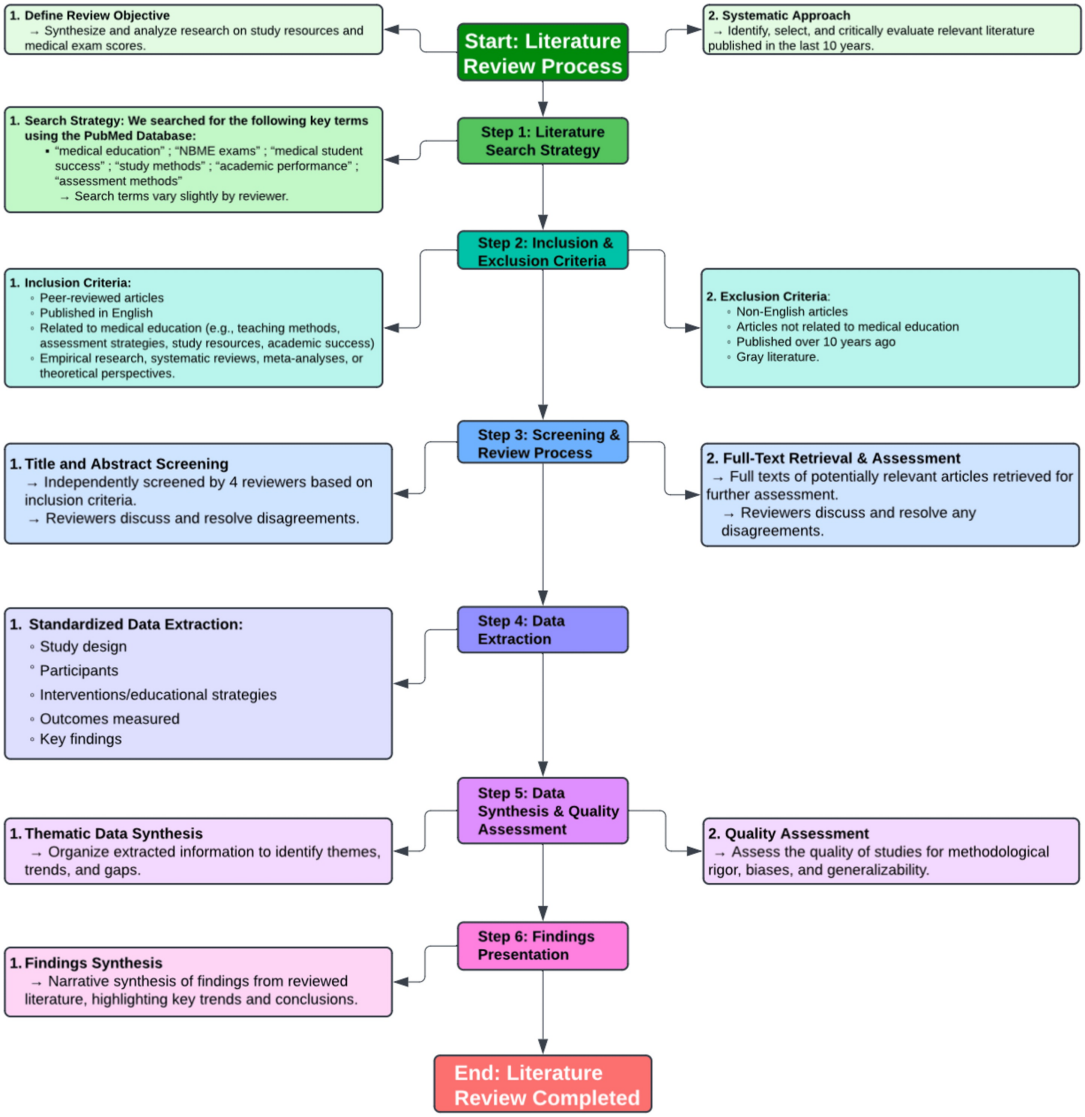
Structured Outline of Our Literature Review Search Strategy Process. NBME: National Board of Medical Examiners

Though there is a vast amount of study resources and strategies available for medical students, the objective of this literature review was to gauge the amount of research that has been done discussing the correlation between study resources and/or methods and exam performance by medical students. This review does not go into detail on the exhaustive list of resources and strategies available. This literature review aims to assess the current research on the relationship between study resources and exam performance. We focus on identifying factors that contribute to higher test scores, drawing insights from recent literature to highlight effective study methods and resources. The search strategy we implemented, which is discussed in more detail in the Literature review methodology section, was very preliminary as the objective was to gain a general idea of what existing literature has looked into regarding this domain of medical education (Figure 1). The scope of this review is narrow and seeks to determine the general consensus of the most available data in the most recent available literature. The search strategy used was a keyword search on the database PubMed. The inclusion criteria focused on articles related to medical education and the journey of medical students from the Medical College Admission Test (MCAT) to Step exams, including study resources. The exclusion criteria included articles that were not within the last 10 years or not part of the medical education domain or study populations with low generalizability.

## Review

Literature review methodology

This literature review strives to synthesize and analyze existing research on various aspects of medical education, particularly looking at the relationship between study resources utilized and standardized medical exam scores among medical students. We followed a systematic approach to identify, select, and critically evaluate relevant literature published within the past 10 years, as outlined in the flow chart in Figure 1. A literature search strategy was used to identify relevant and recent studies within the medical education domain. The electronic database PubMed was systematically searched using key terms. The search terms included combinations of key phrases such as "medical education", “NBME exams", “medical student success”, “medical student study methods”, “medical board study resources”, “academic performance”, and "assessment methods". Key terms slightly varied among the four reviewers working on the review. Studies were included based on predefined criteria, such as peer-reviewed articles published in English, focus on medical education topics such as teaching methods, assessment strategies, study resources used, or academic success of medical students. Studies reporting empirical research, systematic reviews, meta-analyses, or theoretical perspectives were included if found to be relevant. Exclusion criteria included non-English articles, articles not directly related to medical education, articles published over 10 years ago, and gray literature.

Initially, titles and abstracts of identified articles were screened independently by four reviewers against the inclusion criteria. Full texts of potentially relevant articles were then retrieved and further assessed for eligibility. Any disagreements between reviewers were resolved through discussion. Data extraction was performed using a standardized form to capture relevant information from each included study. Key data extracted included study design, participants, interventions or educational strategies evaluated, outcomes measured, and main findings. Data synthesis involved organizing extracted information thematically to identify common themes, trends, and gaps in the literature. The quality of included studies was assessed, and studies were critically appraised for methodological rigor, potential biases, and generalizability of findings. Findings were synthesized and presented descriptively, supported by evidence from the reviewed literature. The focus remained primarily on a narrative synthesis due to the diversity of study designs and outcomes.

Review of literature

Background Perspective on Medical Education

Most students no longer use professional textbooks as their primary study resource, as they now rely on commercial review resources. Though these commercial review resources help with efficient studying, using too many of them may actually reduce their efficacy [15]. While the former fact was significantly associated with poor academic performance, the latter fact had a weak negative correlation with exam scores [15].

Fulton's article [16] on the history of medical education identifies key trends and transformations over the centuries. It explores the evolution from apprenticeship-based learning in ancient times to structured formal education in medieval universities. Pivotal reforms in medical education during the Renaissance and Enlightenment, including the integration of anatomy and scientific principles into medical curricula, were shown to lay the foundation for modern medical education practice [16]. More recent developments such as the shift toward competency-based education, which focuses on assessing practical skills and clinical outcomes, were also discussed. The integration of technology and simulation into medical training to enhance learning and patient safety as well as interdisciplinary education and ethical considerations in medical practice have been the center of recent medical education developments [16].

Recently, more research has been done to examine the impact different types of study resources have on the academic success of medical students. Table 3 lists some studies that have been conducted to investigate.

**Table 3:** Summary of Findings and Implications from Our Review of Recent Studies on Medical Student Performance and Study Methods. CK: Clinical Knowledge; USMLE: United States Medical Licensing Examination; NBME: National Board of Medical Examiners; CBSE: Comprehensive Basic Science Examination; EM: Emergency Medicine; GPA: grade point average

Reference/article	Article conclusions	Implications
[17]	The strategies and resources students utilize when preparing for the Step 2 CK exam and the relationship between these approaches and performance [17]	Looking at how students study for the Step 2 CK exam and how these study methods affect their test scores
[18]	The potential effects of the usage of “medical education commercial-off-the-shelf learning platforms developed for medical education” (MedED-COTS) as a resource for integration in the formal curriculum as well as the effect on students' national licensing exam scores in the USA, as MedED-COTS continue to emerge as a resource for integration in the formal curriculum and potential effects of MedED-COTS [18]	Studying how using commercial medical learning platforms in official courses might impact students' scores on national licensing exams in the USA
[19]	The relationships between academic aptitude, study strategies, and academic performance that may exist [19]	Medical student performance in school might be linked to their abilities, the way they study, and their overall performance
[20]	The relationship between study strategies and performance on a high-stakes medical licensing exam including USMLE Step 1 [20]	Students' study methods can significantly impact their performance on high-stakes medical exams like the USMLE Step 1
[21]	Curriculum intervention can affect NBME Psychiatry Shelf Exam scores [21]	Assessing how curriculum interventions and strategies affect medical student performance can help guide improvement plans for medical students
[22]	Students' performance in medical gross anatomy and the ability to predict USMLE Step 1 exam scores have been suggested based on the idea that a strong understanding of basic medical sciences, like gross anatomy, is crucial for doing well on board exams [22]	Excelling in medical gross anatomy may predict strong performance on the USMLE Step 1 due to its importance in understanding basic sciences
[23]	The role of structured, process-oriented preparation in the reduction of student anxiety for the USMLE Step 1 exam, offering an in-depth look at different preparation methods and their psychological benefits for students [23]. Additionally, the use of practice questions, mock exams, and structured study plans as helpful tools to increase confidence in students, leading to better exam results, has also been investigated [23]	Structured preparation methods, including practice questions and mock exams, can reduce student anxiety and boost confidence, potentially leading to better USMLE Step 1 results
[24]	The role of preparation courses in student preparation for the Step 1 exam as a way to impact medical student academic success [24]	Preparation courses can enhance medical students' academic success by improving their readiness for the Step 1 exam
[25]	The correlation between surgery shelf exam score and the use of popular study materials, the number of study materials used, and the amount of time spent studying throughout the clerkship using a prospective observational method [25]	Medical students' performance on the surgery shelf exam could be influenced by their choice and quantity of study materials and the time they dedicate to studying, suggesting that effective study strategies and resource management are important for improving exam scores
[26]	Whether the NBME CBSE could be used to predict a passing Step 1 score, which could then be used, along with the Medicine Shelf Exam, to predict a passing score on the Step 2 CK exam [26]	If the NBME CBSE and Medicine Shelf Exam can predict Step 1 and Step 2 CK scores, students can use these results to assess their readiness, identify weaknesses, and refine their study strategies to enhance their chances of passing the licensing exams
[27]	The use of various academic indicators in association with Step 2 CK scores to gain an early indication of medical students in relation to its use in academic support [27]	Analyzing various academic indicators can provide early insights into medical students' Step 2 CK scores, which can be useful for tailoring academic support
[28]	Correlations between scores on available fourth-year emergency medicine clerkship exams, via multi-center, prospective paired comparison; particularly the relationship between the National EM M4 Exam score and the Advanced Clinical Examination in Emergency Medicine [28]	The study indicates a relationship between performance on two assessments. This suggests students can use practice exams to gauge progress; thus, implementation as a study strategy can better student performance
[29]	West et al. discussed correlational research that focused on comparing whether studying using practice questions or watching videos is associated with the improvement of students’ test scores on the USMLE Step 1 exam. Data was collected from two separate groups of students from 2022 and 2023. For both cohorts, students who watched videos had a negative correlation with respect to the Step 1 scores while the ones who focused on practice questions had a positive correlation [29]	Students who used practice questions saw improved USMLE Step 1 scores, while those who watched videos had worse scores; provides insight into how different study resources and methods play a role in student performance
[30]	Encandela et al. examine medical students’ test anxiety in relation to the USMLE Step 1 exam, specifically the triggers, effects, and strategies of managing test anxiety for students. The qualitative data collected was from 93 second-year medical students at three separate time intervals. The data was taken before the study period, during the study period, and after the exam. The overall study provides insight into how test anxiety affects students along with the potential interventions [30]	Other factors, such as test anxiety, can affect medical students during their preparation and testing for the USMLE Step 1
[31]	Differences in learning and study strategies among low‐, average‐ and high‐achieving college students with the use of Learning and Study Strategies Inventory (LASSI). The data was collected by gathering 168 undergraduate students from the United Arab Emirates University and splitting them into groups based on their GPA. It was found that low-achieving students scored lowest on all LASSI scales and there was hardly a difference between average- and high-achieving students on any scale [31]	Low-achieving college students have weaker learning and study strategies compared to their average- and high-achieving peers
[32]	The study investigated how learning and study strategies relate to academic performance in first-trimester chiropractic students, using data from 57 students assessed with the LASSI [32]	Learning and study strategies are linked to academic performance
[33]	This article expands on the correlation between the use of study aids and exam performance among second-year medical students. It tests to see if students who create their own study methods or tools tend to perform better than those using study aids already created for them. The study uses questionnaire data connected to students’ exam scores to investigate these relationships further [33]	Medical students who develop their own study methods may perform better on exams compared to those who use pre-made study aids, based on questionnaire data and exam scores
[34]	The article examines self-directed learning in adult education, emphasizing factors like time management, reflective thinking, and critical judgment. It employs a literature review to explore various perspectives and qualities of self-directed learning, offering insights into its multi-faceted nature within adult education contexts [34]	Self-directed learning in adult education includes time management and reflective thinking, with this literature review highlighting its various aspects

As seen in Table 3, the history and evolution of medical education have been shaped by centuries of transformative developments. In the current literature, studies explore diverse aspects of medical education effectiveness. Research examines the impact of study resources on academic success, including the relationship between commercial review resources and exam scores. As outlined by several studies above, strategies and platforms such as medical education commercial-off-the-shelf learning platforms developed for medical education (MedED-COTS) are under study for their integration into curricula and potential effects on national licensing exam performance [17-34]. Furthermore, investigations delve into correlations between study strategies, academic aptitude, and performance on high-stakes exams like the USMLE Step 1. Structured, process-oriented preparation methods have been shown to reduce student anxiety and enhance exam readiness [17,18,21,24]. Additionally, the use of preparation courses and the predictive value of exams like the NBME Comprehensive Basic Science Examination (CBSE) for licensing exams highlight ongoing efforts to optimize medical education outcomes [26].

*Current Trends and Findings of the Reviewed Literature* 

The literature appraised in this review, though all related to medical education and academic success, had different areas of focus. Table 4 lists all key findings found in these recent studies.

**Table 4:** Summary of Research Findings on Study Strategies and Performance Indicators in Medical Education. CK: Clinical Knowledge; NBME: National Board of Medical Examiners; USMLE: United States Medical Licensing Examination; MedED-COTS: medical education commercial-off-the-shelf learning platforms developed for medical education; CBSE: Comprehensive Basic Science Examination; CBSSA: Comprehensive Basic Science Self-Assessment; GPA: grade point average; EM: Emergency Medicine; LASSI: Learning and Study Strategies Inventory

Article	Results of the article	Implications
[17]	Student performance on Step 2 CK, at a single US school, was correlated with performance on previous exams, including school-specific examinations, NBME clerkship shelf exams, and Step 1 based on self-reporting. Two study strategies were positively correlated with Step 2 CK scores in preliminary analyses: completing more working practice questions and the proportion of a question bank completed. Although students used a heterogeneous mix of study strategies and resources, few were positively correlated with examination performance. Notably, students performed better if they focused on working through case-based clinically focused questions. By incorporating the regular review of case-based questions, students engaged in a form of practice testing, which has been found to enhance learning and long-term retention. Studying based on focused content areas did not lead to improved performance. Results confirmed that student performance on clerkship exams was associated with performance on the Step 2 CK exam. Thus, students can reliably use these assessments as indicators of expected performance on Step 2 CK. This affirms the results of a previous study with similar findings. Researchers were unable to identify significant positive associations using specific resources (e.g., texts, videos, study aids, question banks, and practice tests) and performance on the USMLE Step 2 CK exam [17]	Student performance on the Step 2 CK exam was positively correlated with previous exam scores and the use of case-based practice questions, while specific study resources showed limited impact on exam performance, confirming that performance on earlier assessments can reliably predict Step 2 CK outcomes
[18]	Results from a study evaluating MedED-COTS revealed consistent positive correlations between students’ use of question banks and their licensing exam performance. Consistent positive correlations, along with students’ pervasive use and strong theoretical foundations explaining the results, provide evidence for integrating MedED-COTS into medical school curricula [18]	A positive correlation between question bank use and licensing exam performance supports integrating MedED-COTS into medical school curricula, as opposed to traditional lecture-style education
[19]	The results of three regressions indicated that two study skills, time management and self-testing, were generally stronger predictors of first-semester academic performance than aptitude [19]	Prioritizing time management and self-testing as study skills can be more effective in improving academic performance than focusing solely on inherent aptitude
[20]	The results from West et al. [20] indicate that concentration is the only study strategy that appears to be predictive of USMLE Step 1 performance. Since the CBSE and CBSSA averages are also statistically significant predictors of USMLE Step 1 performance, these assessments may possibly offer predictive value of medical student academic success [20]	Focusing on concentration and utilizing predictive assessments like the CBSE and CBSSA can effectively improve USMLE Step 1 performance and guide study strategies. They could offer insight into progress and serve a purpose in improving academic performance
[21]	The average raw Psychiatry Shelf Exam (PSE) score of students offered the review session was 84.53, versus 77.15 for matched controls (p < 0.0001). The effect size for the intervention was 0.89, suggesting that offering a comprehensive review session to third-year medical students three days before their NBME PSE significantly improves their scores. Although performing well on the NBME subject exams is important for clerkship grades, there is also evidence to suggest that studying for shelf exams is beneficial for the long-term consolidation of memory and performance on Step 2 CK [21]. These results can offer insight into how active listening or problem-based learning can play a role in medical education and be a useful study strategy for academic success	Providing comprehensive review sessions significantly improved PSE scores, thus highlighting the value of targeted study interventions, like active listening or problem-based learning, for enhancing long-term academic performance and consolidation of knowledge
[22]	A retrospective review of student grades in gross anatomy and their Step 1 scores suggests that medical schools should place a strong emphasis on teaching gross anatomy to better prepare students for licensing exams [22]. These results underscore the potential of foundational courses predicting exam performance and emphasize the value of rigorous academic support and practice exams	Emphasizing gross anatomy in medical education can significantly enhance student performance on licensing exams, suggesting the importance of foundational courses and rigorous academic support
[23]	Via a combination of qualitative and quantitative methods to assess anxiety levels and exam performance before and after the intervention, results suggest that medical schools should implement structured preparation programs to help students manage anxiety and improve their exam performance. This has important implications for curriculum development, indicating a need for supportive, process-oriented educational frameworks, exploring the need for integrated programs that foster student well-being and academic success [23]	Medical schools should implement structured preparation programs to manage student anxiety to enhance exam performance
[24]	Results suggest that students who used a preparation course scored lower on Step 1 as compared to those who did not; however, these results were not found to be significant when second-year GPA was used as a covariate (p = 0.71). It was concluded that performance on Step 1 was related to medical school performance rather than preparation methods [24]. These results slightly challenge the results of other literature reviewed in this paper, further emphasizing the need for more research in this domain of medical education	Step 1 performance is more closely related to overall medical school performance than to the use of preparation courses, suggesting the impact of study methods on educational outcomes should be further studied
[25]	In a study analyzing how different study resources and study durations affect exam scores during medical clerkships, researchers found that using an online question bank combined with a high-yield review book yielded the highest exam scores (z-score = 6.23). This was followed closely by using the same combination along with one additional resource (z-score = 6.02), two additional resources (z-score = 5.17), or a case-based review textbook (z-score = 4.75). Interestingly, using four different types of resources yielded the highest overall z-score of 6.28. When considering the amount of time spent studying per week, students who studied six to 10 hours per week during the first half of the clerkship achieved the highest z-score of 5.76. For the second half of the clerkship, studying 11 to 15 hours per week resulted in the highest z-score of 6.02, followed by 16 to 20 hours (z-score = 3.97). These findings highlight the significant impact of resource selection and study intensity on medical exam performance [25]	Using a combination of high-yield review materials and online question banks, along with appropriate study durations, significantly enhances exam scores during medical clerkships. Thus, proper resource selection and study intensity play a role in improving medical student academic performance
[26]	A study analyzing the link between CBSE scores and Step 1 passing scores found a significant correlation, suggesting students with a score of at least 66% were more likely to pass Step 1 on their first attempt. When evaluating data for Step 2 CK, the researchers found a significant correlation (r = 0.572, p ≤ 0.001) between the score in the NBME Medicine CSSE and the score in the USMLE Step 2 CK. Based on the data collected, early detection of students at risk for not passing Step 1 and/or Step 2 can provide opportunities for students, schools, and faculty to address knowledge deficiency early on and assess a student’s readiness for USMLE exams [26]	Early assessment using CBSE and NBME Medicine CSSE scores can help identify students at risk of failing Step 1 or Step 2 CK, suggesting that timely interventions to address knowledge gaps can improve exam readiness
[27]	Preclinical mean course exam score and Step 1 score accounted for 56% of the variance in Step 2 CK score. A second series of models included mean preclinical course exam score, Step 1 score, and scores on three NBME subject exams and accounted for 67%-69% of the variance in the Step 2 CK score. The authors created a regression equation and validated it on a different cohort of medical students within a mean of four points (SD = 8). The study’s results showed statistically significant correlations between all predictors and Step 2 CK score [27]	Strong performance in preclinical exams and Step 1, along with additional NBME subject exam scores, can reliably predict success on Step 2 CK, indicating that focusing on these areas can improve educational outcomes
[28]	Pearson’s correlations and linear regression on the NBME raw and scaled scores and EM M4 exams showed a moderate positive correlation for all comparisons, indicating the possibility that practice exams or prior standardized exams can be used as a predicting factor of medical students’ future performance on NBME or USMLE exams, including Step 1 and Step 2 [28]	Practice exams and previous standardized test scores can be useful indicators for predicting future performance on important medical exams like Step 1 and Step 2
[29]	The study concluded that active study methods, such as doing practice questions, are more effective than passive methods, like watching videos. The study suggests a positive correlation between the number of practice questions answered and higher scores on the Step 1 exam. It also found that watching more videos was negatively correlated with Step 1 exam scores, proving that active learning strategies are essential to better performance on the Step 1 exam [29]	Active study methods, such as practice questions or question banks, are more effective than passive methods like watching videos, with a positive correlation between practice question volume and higher Step 1 exam scores
[30]	It was found that test anxiety negatively affects the emotional and cognitive aspects of students and can also impact them physically, supporting the idea that resources or interventions that are aimed to reduce anxiety will enhance overall student success and exam performance [30]	Reducing test anxiety through targeted interventions can improve both emotional and cognitive aspects of student performance, leading to better exam outcomes for medical students
[31]	The results highlighted the need for motivation in distinguishing low-achieving students from high-achieving ones. The findings of this study imply that educational interventions should place their focus on bettering and increasing motivation along with study strategies, mostly for low-achieving students. Adding motivational support systems and study methods for students could potentially lead to the enhancement of student performance [31]	Enhancing motivation and study strategies, especially for low-achieving students, can improve academic performance
[32]	The data found in this study indicates that students with a higher GPA scored better on the LASSI subtests and on effort-related activities and goal-oriented factors, thus proving the importance of motivational practices and strategies. The t-tests used in this study were to compare high and low GPA from students, and it was found that using this method gave results that indicated the difference learning strategies make [32]	Students with higher GPAs excelled in LASSI subtests and effort-related activities, highlighting the impact of motivational practices and learning strategies
[33]	The data collected in this study supports that while most students use study aids to review information, the students who performed best were those who used pre-existing study tools. The study suggests that schools should encourage individual studying habits and strategies particularly for those students on different levels of exam performance. It highlights the importance of effective study techniques that are tailored to the students’ needs and learning preferences [33]	While most students use study aids, those who performed best used pre-existing tools, indicating that schools should promote personalized study strategies tailored to individual learning preferences and performance levels
[34]	The findings of this research display the need for educators to understand adult learners and support them when it comes to self-direction. Adapting techniques to accommodate them could potentially enhance their learning outcomes [34]	The importance of educators understanding and supporting self-directed learning in medical students could improve learning outcomes

Recent studies in medical education reveal several key findings and common themes, as seen in Table 4. Firstly, student performance on Step 2 CK exams shows correlations with prior academic performance, including results from school-specific exams, NBME clerkship shelf exams, and Step 1 scores [17,20]. Notably, completing more practice questions and utilizing question banks are positively linked to higher Step 2 CK scores, reflecting effective study strategies [28]. However, many other study resources showed inconsistent correlations with exam performance, emphasizing the need for further research into optimal study methods. For instance, Zhang et al. [24] determined that performance on Step 1 was related to medical school performance rather than preparation methods [24]. These results slightly challenge the results of other literature reviewed in this paper, further emphasizing the need for more research in this domain of medical education. On another note, studies also highlight the effectiveness of case-based clinically focused questions in improving exam scores, suggesting that practice testing enhances learning and retention. Additionally, findings indicate that performance on clerkship exams reliably predicts Step 2 CK outcomes, underscoring the predictive value of these assessments [17]. Research on MedED-COTS supports its integration into curricula, as question bank use consistently correlates with licensing exam performance [18]. Moreover, factors such as time management and self-testing skills emerge as stronger predictors of academic performance than innate aptitude. Conversely, specific study strategies like content-focused studying do not consistently improve exam results. Furthermore, structured preparation programs and comprehensive review sessions have been shown to significantly boost exam scores, highlighting the importance of educational interventions in reducing student anxiety and enhancing academic success. Lastly, correlations between preclinical course scores, Step 1 performance, and NBME subject exams collectively account for a substantial variance in Step 2 CK scores, reinforcing the importance of foundational knowledge and ongoing assessment in medical education [20]. These studies underscore the complex interplay of study resources, strategies, and assessment tools in shaping medical student outcomes, prompting ongoing exploration into effective educational practices. Additionally, some of the study results highlight the significance of motivation in distinguishing between high- and low-achieving students, suggesting educational interventions should focus on enhancing both motivation and study strategies, particularly for students with lower academic performance [31].

Areas of Agreement and Disagreement in the Reviewed Literature

The investigation of MedED-COTS as a study strategy in relation to licensing exam performance can enhance the coaching of individuals to improve their performance on medical examinations [18]. Assessing information regarding hours spent studying and resources used by medical students can also provide useful insights to better teach students how to prepare for medical examinations [25]. Studies like these that look into a range of preparation methods will assist medical students and faculty when formulating study schedules when preparing for an exam, such as Step 1 [24]. Teaching students to practice and utilize certain techniques aimed at improving concentration skills when preparing and taking exams may result in higher Step 1 scores. Understanding the relationship between learning and study strategies and performance on high-stakes exams can inform exam preparation and performance, educational intervention, and curriculum planning [30]. A consensus of prior statistical analysis shows a significant correlation, highlighting the importance of solid foundational knowledge, supporting the impact educational practices have on exam success and overall student performance [22].

Current studies generally agree that the types and amounts of study resources used by medical students correlate with exam performance, particularly for high-stakes exams like Step 1 of the USMLE [25]. Some studies emphasize the importance of comprehensive preparation methods [24], while others highlight the effectiveness of specific study techniques and concentration skills [30] in improving exam scores. Overall, there is consensus that a strong foundational knowledge base is crucial for success on medical licensing exams [22], underscoring the ongoing need for evidence-based educational practices to support student performance.

Gaps in the Reviewed Literature

Although the importance of preparing for NBME and USMLE examinations has been established, there remains a high degree of variability in the methods and strategies that medical students and educators use in preparation for academic success. More studies should be conducted to evaluate the use of resources on NBME Step scores by evaluating the correlation between scores and specific resources used by medical students [25,26]. Studies have found that most students on their psychiatry clerkship rely heavily on “step” or “prep” books and that these review materials “did not demonstrate significance as a superior preparation resource for the psychiatry subject exam (PSE)” [21], warranting further exploration into alternative effective study materials. While prior research has examined the utility of various academic indicators to predict student performance on the USMLE exams, no significant scholarly effort has been conducted to specifically investigate students' Step 2 CK study approaches. Furthermore, due to the limited number of studies published on either the use or integration of MedED-COTS, the current review of literature can only serve as guidance for future research as opposed to defining the correlations that may exist [18]. Other avenues of research may be in the investigation of the efficacy of a student-initiated curriculum as a form of improvement in medical student academic success [34,35]. Different resources and different practice exams can be used to assess a student’s understanding of the material and help prepare students for Step exams: a comparison of the correlation between multiple resources and how scores correlate to help assess a student’s knowledge base [28]. This domain of medical education research can help establish effective study resources and strategies that can be used as an early intervention to improve USMLE Step exam scores for medical students [27,36,37].

## Conclusions

Our review explores current literature on the relationship between medical school academic performance and USMLE Step scores, with a particular focus on whether effective study methods-such as practice questions and spaced repetition-impact these outcomes. This review suggests the relationship between medical school academic performance and USMLE Step scores is shaped by a range of factors, with prior achievements, such as medical school exams, NBME clerkship shelf exams, and Step 1 scores, playing a significant role in predicting outcomes. Effective study strategies, particularly the use of practice questions and question banks, suggest a positive impact on higher Step 2 CK scores, though the effectiveness of other resources remains less clear. Performance on clerkship exams also appears to be a strong predictor of Step 2 CK success. Additionally, time management and self-testing are found to be more reliable indicators of academic success than innate aptitude, while content-focused strategies tend to show mixed results. Structured preparation programs, which help manage anxiety, were also found to contribute to improved exam performance. While these findings highlight important trends between study resources and exam performance, gaps remain in understanding the full range of effective study methods and the role of external factors. We recommend that future research should focus on curriculum improvements, the comparative efficacy of various study resources, and management strategies of external factors affecting academic performance to refine preparation methods and enhance student performance outcomes in medical education.
